# Estimating the distribution of the window period for recent HIV infections: A comparison of statistical methods

**DOI:** 10.1002/sim.3941

**Published:** 2010-12-16

**Authors:** Michael Sweeting, Daniela De Angelis, John Parry, Barbara Suligoi

**Affiliations:** a MRC Biostatistics Unit, Institute of Public HealthRobinson Way, Cambridge CB2 0SR, U.K; b Health Protection Agency Centre for Infections61 Colindale Avenue, London NW9 5EQ, U.K; c National AIDS Unit, Department of Infectious Diseases, Istituto Superiore di SanitáViale Regina Elena 299, 00161 Rome, Italy

**Keywords:** HIV incidence, biomarker, mixed-effects models, window period

## Abstract

In the past few years a number of antibody biomarkers have been developed to distinguish between recent and established Human Immunodeficiency Virus (HIV) infection. Typically, a specific threshold/cut-off of the biomarker is chosen, values below which are indicative of *recent infections*. Such biomarkers have attracted considerable interest as the basis for incidence estimation using a cross-sectional sample. An estimate of HIV incidence can be obtained from the prevalence of *recent infection*, as measured in the sample, and knowledge of the time spent in the *recent infection* state, known as the window period. However, such calculations are based on a number of assumptions concerning the distribution of the window period. We compare two statistical methods for estimating the mean and distribution of a window period using data on repeated measurements of an antibody biomarker from a cohort of HIV seroconverters. The methods account for the interval-censored nature of both the date of seroconversion and the date of crossing a specific threshold. We illustrate the methods using repeated measurements of the Avidity Index (AI) and make recommendations about the choice of threshold for this biomarker so that the resulting window period satisfies the assumptions for incidence estimation. Copyright © 2010 John Wiley & Sons, Ltd.

## 1. Introduction

Incidence estimation has long been the holy grail of Human Immunodeficiency Virus (HIV) epidemiological research. Estimates of incidence are needed to monitor ongoing transmission, to evaluate interventions aimed to reduce transmission and to plan resource allocation for prevention. Cohort studies that follow-up uninfected individuals, the gold standard for incidence estimation, are expensive to run and can be subject to observational biases due to selection and follow-up adherence 1. Thus, historically, much effort has been put into the development of indirect methods of estimation 2–6. Methods based on ‘snapshot’, or cross-sectional, sampling 1, 5, 6 have attracted considerable interest in recent years as laboratory methods, based on characteristics of the antibody response soon after infection, are being continuously developed to identify recent infections.

The idea underlying these methods, or at least their simplified version, is as follows. Let *d* be the date on which a cross-sectional survey is conducted and the sampled individuals are tested for HIV and classified as negative or positive and, among the positive, as *recently infected* or not according to the measured level of a chosen biomarker. The prevalence of *recent infection* *P*(*d*) at date *d* can be expressed in terms of the incidence density of HIV at time *t*, *I*(*t*) as


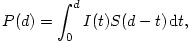
(1)

where *S*(*t*) is the survival function of the time spent in the *recent infection* state, the so-called *window period*. HIV incidence *I*(*t*) is commonly estimated from (1) under two assumptions. First, there exists a maximum window period *w*_*m*_ such that *S*(*w*_*m*_) = 0, and second the incidence is constant over the past *w*_*m*_ years, that is over the calendar period [*d*−*w*_*m*_, *d*]. Under these assumption, Equation (1) simplifies as follows:


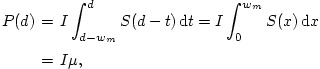
(2)

where µ is the mean of the window period distribution (see 1 for more details). The problem of estimating *I* then becomes that of using a cross-sectional (random) sample to estimate the prevalence of those recently infected, and to acquire the necessary knowledge of µ. Owing to the assumptions underlying Equation (2) it is, therefore, undesirable for *w*_*m*_ to be too large and hence the distribution of the window period to have a long tail.

In the last 10 years a number of assays have been proposed to detect recent infections. The original procedure involved testing individuals using Sensitive/Less Sensitive (S/LS) commercial antibody assays (e.g. 3A11-LS, LS EIA), in order to detect differential HIV titre 7. More recently a biomarker has been proposed based on the principle that antibodies produced early after infection bind less strongly to the antigen than those produced in established infection 8. The *avidity* of the antibodies to bind to the antigen can be measured using the Avidity Index (AI). The AI is calculated by dividing the sample-to-cutoff (S/CO) ratio from a low-avidity sample treated with guanidine by the S/CO ratio from a control sample, more details of which can be found in 9. For early infection, weak binding causes the level of antibodies in the treated sample to be less than that in the control, and hence the AI takes values less than one. For more established infection, antibody levels in the two samples are similar and hence the AI approaches a value of one. Conditionally on the choice of a specific threshold, commonly 0.8, individuals with measured AI below the threshold are classified as *recently infected* and the window period is the time spent below the chosen threshold.

It is clear that the window period is a fundamental ingredient in the estimation of HIV incidence. It depends on the rate of antibody response and hence can vary considerably between individuals. By raising or lowering the associated threshold, the window period can be lengthened or shortened, respectively. If it is too short very few individuals are classified as *recently infected*, resulting in a loss of precision for incidence estimation; too long and the assumption of a constant incidence is no longer viable. Hence knowledge about the distribution of the window period, not just its mean, is essential. Despite this, it is commonly the case that a threshold is chosen based on the diagnostic accuracy of classifying individuals as *recently infected*, where true recency is defined as a certain period post-seroconversion, rather than based on the resulting distribution of the window period.

The aim of this paper is to illustrate two statistical methods for estimating the distribution of threshold-specific window periods. The first method (Section 2) implements a doubly censored survival analysis approach to obtain a non-parametric estimate of the window period distribution. The second method (Section 3) is based on modelling the individual growth curves of the biomarker using mixed-effects models, and inverting the functional relationship to obtain estimates of the window period distribution. In Section 4, we apply the methods to data from a cohort of HIV infected individuals. For each individual AI measurements are available longitudinally and the dates of the last negative and first positive HIV antibody test are known. Finally in Section 5 we make recommendations for the choice of threshold associated with the AI assay so that the resulting window period distribution is likely to satisfy the assumptions required for Equation (2).

## 2. Estimation using non-parametric survival analysis

**Figure 1:**
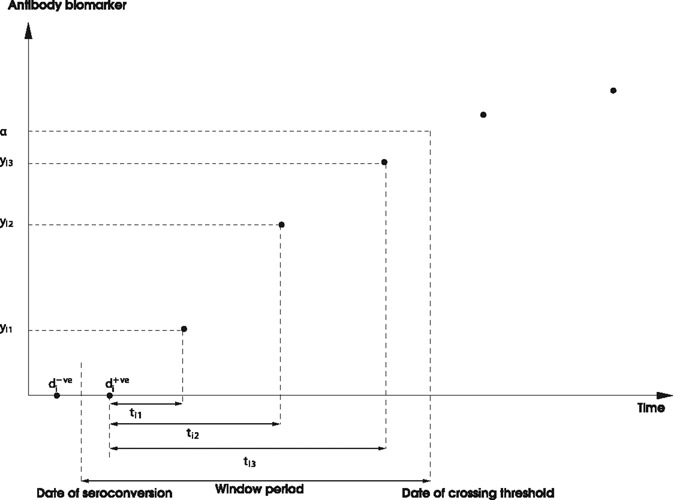
Typical data available from an individual with repeated biomarker measurements. The window period is defined as the unknown time from seroconversion to crossing the threshold, α.

Suppose data on *n* individuals consist of the dates of the last negative and the first positive test results, as established using the standard enzyme immunoassay, and repeated measurements of an antibody biomarker. For individual *i* we have dates 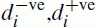 and a sequence of *m*_*i*_ measurements *y*_*ij*_, taken at times *t*_*ij*_, measured from the first positive test date 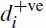. Note that the interval (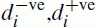] is the (seroconversion) interval within which individual *i* has seroconverted. The aim is, for a given biomarker threshold α, to estimate the distribution of the time from seroconversion till the biomarker crosses α (Figure 1).

Let *X* and *Z* denote the unknown date of seroconversion and crossing the threshold, respectively. For individual *i* we know that 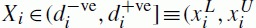]. Further, if the growth of the biomarker is assumed to be monotonically increasing with no measurement error, then we also know that 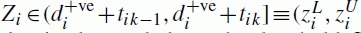], where the *k*th measurement is the first time after testing positive that the biomarker is observed above the threshold. If an individual is observed to be above the threshold on their first measurement, then the only information is that *Z*_*i*_∈(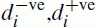 + *t*_*i*1_]. Conversely, if an individual is never observed to be above the threshold then *Z*_*i*_ is right censored and 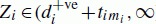].

**Figure 2:**
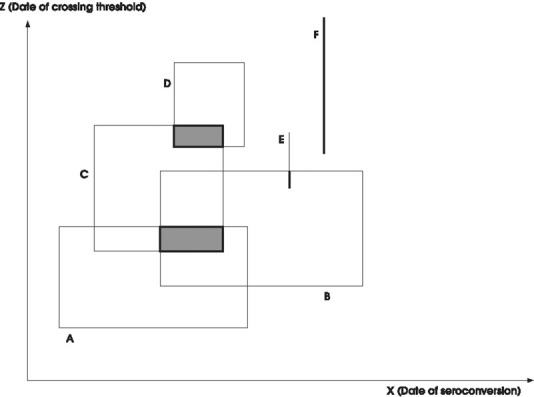
Illustration of 6 individuals with unknown date of seroconversion and unknown date of crossing threshold. Each rectangle represents the region in which the point (*x, z*) is known to lie for that individual. The shaded areas show the regions where all the mass of the NPMLE estimate lies.

The window period for threshold αis defined as *T* = *Z*−*X*. To estimate the distribution of the window period correctly the bivariate density for (*X, Z*) needs to be modelled from which the distribution for *T* can be derived. Similar techniques have been used to estimate the time from seroconversion to AIDS 10, 11. A univariate survival analysis of the interval-censored data *T*∈(*z*^*L*^−*x*^*U*^, *z*^*U*^−*x*^*L*^] assumes an incorrect likelihood and hence such an approach should be avoided 12, although Reich *et al.* 13 find that such an approach can provide reliable estimates of the median. For each individual, the pair (*x, z*) are known to lie within the region 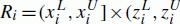] and hence the likelihood of the observations for *n* individuals is


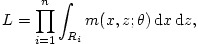


where *m*(*x, z*;θ) is the joint density of (*X, Z*) given parameters θ. To obtain the non-parametric maximum likelihood estimate (NPMLE) of *m*(*x, z*), De Gruttola and Lagakos 10 generalized the self-consistency algorithm of Turnbull 14 for singly censored univariate data. As an example, Figure 2 shows rectangles *R*_*i*_ from six fictional individuals to illustrate where the NPMLE assigns mass. The shaded regions with bold outline show where the NPMLE mass lies. Gentleman and Vandal 12 used concepts from graph theory to show that all the mass associated with the NPMLE lie within the maximal intersections of the rectangles *R*_1_, …, *R*_*n*_.

One difficulty for bivariate interval-censored data is that the NPMLE estimate may be non-unique. Representational non-uniqueness occurs whenever the maximal intersections of the rectangles are not points, as is the case in Figure 2. The NPMLE does not indicate how the mass within each intersection should be assigned. This can be extremely problematic if much of the data are right censored in one dimension, as occurs with line segment *F*. The probability mass must then be assigned over an infinite line segment. To overcome this non-uniqueness one must make some parametric assumption. The mass could be distributed parametrically over the intersections (*e.g* uniformally), or all mass could be placed at a single point, for example at the midpoint of each region. For estimation of the window period *T* = *Z*−*X* one useful approach is to assign all the mass to either the bottom right-hand corner or the top left-hand corner of each maximal intersection region. Doing this allows us to obtain an upper and lower limit, respectively, for the cumulative distribution function (CDF) of *T*. Such an approach is demonstrated in Section 4 where we obtain upper and lower limits for the window period CDF from an HIV cohort with longitudinal AI measurements.

## 3. Modelling the growth of the biomarker

The survival analysis approach presented in Section 2 does not utilize all the repeated biomarker measurements that are available. Furthermore, it does not allow for measurement error in the biomarker process, since the date of crossing the threshold is assumed to lie between the last date *observed* to be below and the first date *observed* to be above the threshold. In addition, the NPMLE is likely to be non-unique and hence a parametric assumption must be made. This warrants an alternative parametric approach where the growth of the biomarker is modelled.

To illustrate, we develop a growth model for the AI, but similar parametric models can be developed for other antibody biomarkers. It shall be assumed, with some biological rationale, that the underlying (latent) antibody response to HIV infection increases monotonically over a period of time. Furthermore, for established infection we assume the AI to approach a value of one. These two observations lead us to consider a non-linear monotonically increasing function with an asymptote in which to model the growth of the AI over time. Let 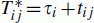 be the unknown time from seroconversion to the *j*th measurement, where τ_*i*_ is the unknown time from seroconversion to the first positive date for the *i*th individual. Then the growth of the AI can be modelled using the following mixed-effects model:


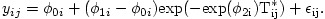
(3)

This three-parameter non-linear function is monotonically increasing, and approaches an asymptote, ϕ_0*i*_, as time tends to infinity. The parameter ϕ_1*i*_ is the intercept and is interpreted as the value of the AI at seroconversion, and ϕ_2*i*_ is the logarithm of the rate constant. The 
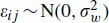
 are normally distributed error terms. The parameters, ϕ_0*i*_, ϕ_1*i*_, and ϕ_2*i*_ are specified as random-effects, to allow between person variability although in Section 4 we shall consider whether each can be modelled as a fixed-effect.

The random-effects are modelled using a multivariate Normal distribution, which allows us to borrow strength between individuals, (ϕ_0*i*_, ϕ_1*i*_, ϕ_2*i*_)^*T*^∼N_3_((µ_0_, µ_1_, µ_2_)^*T*^, Σ_*b*_), where Σ_*b*_ is an unstructured variance–covariance matrix. The final requirement is the specification of τ_*i*_ for each individual. One assumption is that the seroconversion date is exactly at the midpoint of the seroconversion interval, and hence 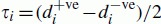. This however disregards any uncertainty about the date of seroconversion. Hence we will label the model using this assumption as the *naïve* model. More realistically a distribution can be placed on τ_*i*_, and without further knowledge about testing strategies or AI measurements, a sensible *a priori* belief is that τ_*i*_∼Uniform(0, 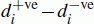) (i.e. that seroconversion is equally likely to occur at any time during the seroconversion interval). We shall label this model the *uniform prior* model. Since our beliefs about the distribution of τ_*i*_ may change once we model the growth of the AI measurements, it is intuitive to perform such analyses from a Bayesian viewpoint. In Section 4 we shall investigate the use of both the *naïve* and *uniform prior* models in this framework.

### 3.1. Inverse prediction

The aim is to estimate the window period, i.e. the time from seroconversion to crossing a specified threshold, α, using the fitted parametric model. This can be achieved using an inverse prediction technique. For a threshold α, the associated window period for individual *i*, 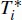 (α), can be expressed as a function of the random-effects for patient *i* using Equation (3):


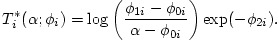
(4)

Note that, since we are interested in the time to truly crossing threshold α, this prediction does not include the measurement error term. Using the relation (4) the posterior distribution of the window period for individual *i*, *p*(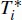
(α;ϕ_*i*_)∣**y**_***i***_), can be easily derived using Markov chain Monte Carlo (MCMC) methods, together with the posterior distribution of any function of the 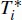 (α)'s. Specifically, the average window period in the given sample can be calculated as 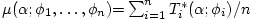 and its distribution obtained over the MCMC iterations. A further use of the parametric model is to obtain a predictive distribution of the window period for a *new* individual (an out-of-sample prediction). This is achieved by first sampling new random-effects ϕ_0new_, ϕ_1new_, and ϕ_2new_ from the posterior distribution of the random-effects superpopulation. Then for each realization we can calculate the associated window period


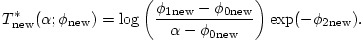


The mean, median, and percentiles for this out-of-sample prediction can again be easily estimated from the MCMC sample. The prediction is for a generic new individual and hence automatically accounts for between individual variability.

## 4. Illustration: repeated AI measurements from a cohort of HIV seroconverters

### 4.1. Data

**Figure 3:**
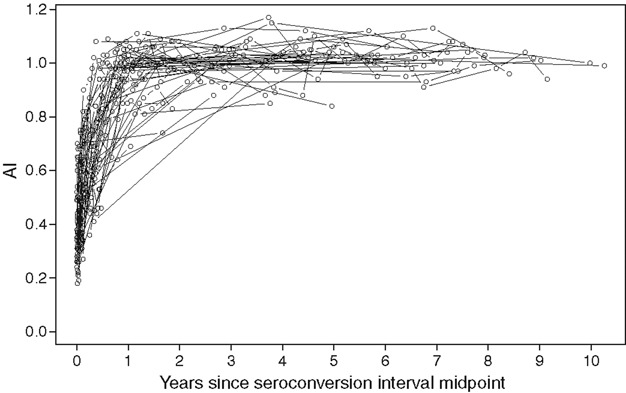
Data from 103 individuals showing the growth of the AI, where the time origin is defined as the midpoint of the seroconversion interval.

Data are available on 175 HIV seroconverters consisting of the last negative and the first positive test dates, using a standard immunoassay (Abbott AxSYM HIV 1/2 gO), together with a series of AI measurements post HIV diagnosis. All individuals were identified as first-time HIV-positives in voluntary counseling and testing centres located in 4 hospitals (2 in Rome, 1 in Turin, and 1 in Brescia). Of these individuals, 72 had one AI measurement and therefore provide no information on the growth rate of AI. Hence this analysis uses data from the 103 individuals with two or more AI measurements. There are on average four AI measurements per person, with the maximum number of measurements being 10, and the minimum 2. The time between the last negative and the first positive test (the seroconversion interval) is on average 3.6 months, but there is considerable variation between patients, with intervals ranging from 2 days to 18 months. The mean value of the first AI measurement after diagnosis is 0.60, but again there is considerable variation (range 0.19, 1.09), reflecting the fact that individuals are diagnosed at different periods post seroconversion. Individual growth patterns of AI are shown in Figure 3, where time is plotted from the midpoint of the seroconversion interval. Most of the AI growth occurs within the first 12 months of the seroconversion midpoint, and an asymptote close to 1 is soon reached. However, there is a large variation in measurements taken close to the seroconversion midpoint. This could be due to the natural variation between individuals, and/or because the exact date of seroconversion is unknown.

### 4.2. Non-parametric window period estimation

**Figure 4:**
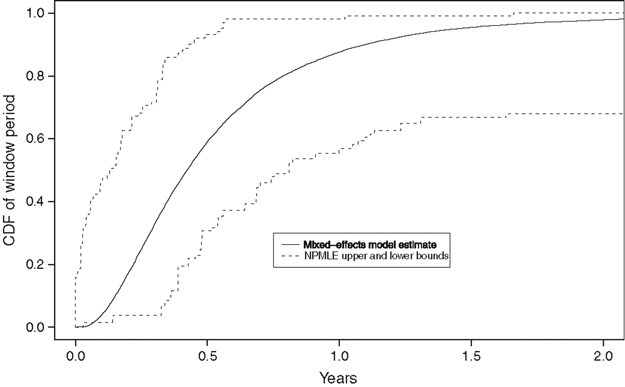
Cumulative distribution function of the predicted window period for a new individual for an AI threshold of 0.8. The solid line shows the distribution from the non-linear mixed-effects model. The dotted lines show the upper and lower bounds as calculated from the NPMLE.

The date of crossing the 0.8 threshold is right censored for many individuals, especially those who seroconverted after 2002. This causes non-uniqueness in the NPMLE as discussed in Section 2. Using the MLEcens package in R 15 we calculate the upper and lower bounds of the CDF for the window period. These are shown as dotted lines in Figure 4.Clearly, inferences based on these non-parametric limits are of little practical use, suggesting that the growth of the AI should be modelled parametrically.

### 4.3. Longitudinal modelling

To fit the growth models we use a Bayesian approach implemented through MCMC in WinBUGS 16, code for which is available from the authors on request. Non-informative Gaussian priors are placed on the means µ_0_, µ_1_, and µ_2_, while 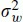 is given an inverse-Gamma(0.001, 0.001) prior. To ensure that Σ_*b*_ is positive-definite, an inverse-Wishart prior distribution is used with degrees of freedom equal to one plus the dimension of Σ_*b*_. This has the effect of placing a uniform distribution on each of the correlation parameters 17. Results (not shown) from the *uniform prior* non-linear mixed-effects model indicate that even after accounting for unknown seroconversion date there is still considerable evidence that intercepts should be treated as random-effects (fixed effect vs random-effects deviance information criterion (DIC) 18, −797 vs −892). There is also variation in the individual rates of growth, but no evidence for random asymptotes (fixed vs random DIC, −892 vs −874). Table I shows estimates from the *naïve* and *uniform prior* non-linear mixed-effects models treating the asymptote as a fixed effect. The asymptote is estimated to be just over 1 for both models and the population mean intercept, i.e. the value of the AI at seroconversion, is approximately 0.35. There is a slight difference between the models in the population mean estimate of the log-rate parameter: on average the rate of growth is less in the *uniform prior* model. However, the main difference between the two models is in the estimate of the between-subject standard deviations (SD). The between-subject SD for the intercept is lower in the *uniform prior* model, whereas the SD for the log-rates is slightly higher. The reduced variability in the intercepts is explained by properly accounting for the unknown date of seroconversion, allowing for a more homogenous population at seroconversion. The slightly increased variability in the log-rates gives a more honest reflection of our uncertainty in the growth rate of the AI when date of seroconversion is unknown. The *uniform prior* model also has a lower DIC suggesting a better model for predictive accuracy.

**Table I:** Parameter estimates from the *naïve* and *uniform prior* non-linear mixed-effects models

Parameter	Naïve model Posterior median (SD)	Uniform prior model Posterior median (SD)
*Population means*		
Asymptote µ_0_	1.017 (0.007)	1.016 (0.006)
Intercept µ_1_	0.346 (0.023)	0.349 (0.021)
Log-rate µ_2_	0.934 (0.119)	0.964 (0.122)
*Between individual standard deviations*		
Intercept	0.145 (0.021)	0.125 (0.019)
Log-rate	0.835 (0.109)	0.860 (0.110)
Correlation between intercepts and log-rates	−0.53 (0.14)	−0.59 (0.14)
Within-individual standard deviation	0.076 (0.003)	0.074 (0.003)
Posterior mean deviance	−967.3	−991.6
Effective no. of parameters	96.7	99.6
Deviance information criterion	−870.6	−892.0

An interesting question is whether it is possible to use the information on the longitudinal values of the biomarker to infer the unknown seroconversion date. This can be assessed by looking at the difference between the prior and posterior distributions for τ_*i*_. If any learning about τ_*i*_ is taking place, we would expect the difference between the prior and posterior SD to be positive, and the difference between prior and posterior means to give information about the direction of any shift. For the majority of individuals (66 per cent) the difference between the prior and posterior mean for τ_*i*_ is small (within 5 per cent of the length of the seroconversion period), and for these individuals the prior and posterior SDs are very similar suggesting that little is learnt about the seroconversion time. However, for 17 of the individuals the prior SD is noticeably greater than the posterior (over 5 per cent of the length of the seroconversion interval). This implies that it is possible to learn about the seroconversion time. For these individuals we estimate the posterior mean seroconversion time to be closer to the last negative test date (i.e. towards the beginning of the seroconversion interval) for 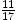 (65 per cent) of the subjects.

Predictions for the in-sample and out-of-sample window period distributions are given in Table II.In this application both models provide almost identical predictions of these distributions. The estimated mean time for the sample to cross the 0.8 threshold is 202 days (95 per cent CrI 174, 245). However, the distribution of the window period is right-skewed since the mean is considerably larger than the median. The estimated 90th percentile of the window period distribution is estimated with high imprecision for this sample of individuals. For a threshold of 0.8, the model predicts that only 88 per cent of new individuals will cross the threshold within 12 months of seroconversion. This compares with 93 per cent for a threshold of 0.75, 96 per cent for a threshold of 0.7, and 99 per cent for a threshold of 0.6. The complete CDF for a threshold of 0.8 is shown as the solid line in Figure 4. As expected, the prediction of the CDF from this parametric model lies within the non-parametric bounds.

**Table II:** In-sample window period and out-of-sample probabilities of reaching threshold within given time periods, for the *naïve* and *uniform prior* models

		In-sample window period, days*			Predicted out-of-sample probability of reaching threshold			
			
Model	Threshold	Mean	Median	90th percentile	0–3 months	0–6 months	0–9 months	0–12 months
*Naïve* model	0.60	72	59	145	0.73	0.94	0.98	1.00
		(63, 86)	(49, 68)	(117, 184)				
	0.70	125	100	244	0.46	0.80	0.92	0.96
		(109, 149)	(86, 114)	(197, 314)				
	0.75	160	125	309	0.34	0.70	0.86	0.93
		(139, 191)	(108, 144)	(249, 405)				
	0.80	203	156	391	0.24	0.58	0.78	0.88
		(175, 244)	(135, 180)	(312, 520)				
*Uniform prior* model	0.60	71	56	139	0.75	0.95	0.98	0.99
		(61, 85)	(46, 66)	(112, 180)				
	0.70	125	97	242	0.46	0.81	0.92	0.96
		(108, 149)	(82, 112)	(193, 319)				
	0.75	160	122	311	0.34	0.70	0.86	0.93
		(138, 192)	(104, 141)	(246, 413)				
	0.80	202	152	395	0.25	0.59	0.78	0.88
		(174, 245)	(129, 176)	(310, 529)				

* Posterior median and 95 per cent credible intervals presented for each quantity.

## 5. Discussion

In this paper we have estimated the distribution of a window period using two statistical methods. For the AI we estimate the window period associated with a number of different thresholds. A threshold of 0.8 has previously been suggested as a cut-off to classify individuals as recently infected, based on sensitivity and specificity of the biomarker 6 months after the midpoint of the seroconversion interval 8, 19. We find such a threshold to be associated with a mean window period of 202 days (95 per cent CrI 174, 245). The probability that the window period is longer than one year is not insubstantial, estimated to be 12 per cent. For a period of three years this probability drops to less than 1 per cent. A threshold of 0.75 or 0.7 may be an alternative choice for incidence estimation, since the probability of the window period being greater than one year is low, at 7 and 4 per cent, respectively. For Equation (2) to hold, a long-tailed distribution for the window period is undesirable and can violate the assumption of a constant incidence over the duration of the window period 1. Indeed with all incidence assays the full distribution of the window period, and not just the mean, should be considered and explored before use in incidence estimation, a fact that is often ignored.

The use of a mixed-effects model to describe the growth of an antibody assay while incorporating uncertainty associated with the seroconversion time is novel. Many previous analyses have assumed seroconversion to be at the midpoint of the seroconversion interval 8, 20–22. We have shown that, for our sample, this *naïve* approach and the *uniform prior* approach result in almost identical predictions of the window period. This could be due to the relatively short duration between the last negative and the first positive tests in our cohort or the fact that the prior mean for the *uniform prior* model is the midpoint of the seroconversion interval. However, for a cohort with longer seroconversion intervals the *uniform prior* model would be more realistic, so that the uncertainty in the data is properly accounted for. This model reflects our *a priori* belief that seroconversion is equally likely to occur anywhere between the last negative and the first positive test. Other choices of distribution can easily be incorporated to reflect different *a priori* beliefs.

By comparing prior and posterior distributions it is clear that for some individuals we have been able to learn about their date of seroconversion from their longitudinal series of AI measurements. However, for other individuals very little information about their date of seroconversion can be gleaned. The mixed-effects model could therefore in theory be used to predict the date of seroconversion for a new individual given information on their AI measurements and seroconversion interval.

The estimates of the mean window period presented here for the AI now require validation from other studies of seroconverters. It is imperative that selection biases are avoided when recruiting such cohorts in order for the distribution of the window period to be as representative as possible. We have found considerable variation in the growth of the AI between individuals, although the heterogeneity could not be further investigated since no covariate information about these individuals was available. However, a previous study has found no effect of antiretroviral treatment, protease inhibitors, sex, or age on the growth rate of the AI, suggesting that it is potentially a robust biomarker 8. Nevertheless, further research into the characteristics of the AI is warranted to enable its use for incidence estimation.

## References

[1] Brookmeyer R (2009). Should biomarker estimates of HIV incidence be adjusted?. AIDS.

[2] Brookmeyer R, Gail MH (1988). A method for obtaining short-term projections and lower bounds on the size of the AIDS epidemic. Journal of the American Statistical Association.

[3] Isham V (1988). Mathematical-modeling of the transmission dynamics of HIV infection and AIDS—a review. Journal of the Royal Statistical Society Series A—Statistics in Society.

[4] Ades AE, Medley GF (1994). Estimates of disease incidence in women based on antenatal or neonatal seroprevalence data: HIV in New York City. Statistics in Medicine.

[5] Kaplan E (1997). Snapshot samples. Socio-Economic Planning Sciences.

[6] Karon JM, Song R, Brookmeyer R, Kaplan EH, Hall HI (2008). Estimating HIV incidence in the United States from HIV/AIDS surveillance data and biomarker HIV test results. Statistics in Medicine.

[7] Janssen RS, Satten GA, Stramer SL, Rawal BD, O'Brien TR, Weiblen BJ, Hecht FM, Jack N, Cleghorn FR, Kahn JO, Chesney MA, Busch MP (1998). New testing strategy to detect early HIV-1 infection for use in incidence estimates and for clinical and prevention purposes. Journal of the American Medical Association.

[8] Suligoi B, Massi M, Galli C, Sciandra M, Di Sora F, Pezzotti P, Recchia O, Montella F, Sinicco A, Rezza G (2003). Identifying recent HIV infections using the avidity index and an automated enzyme immunoassay. Journal of Acquired Immune Deficiency Syndromes.

[9] Chawla A, Murphy G, Donnelly C, Booth CL, Johnson M, Parry JV, PhillipS A, Geretti AM (2007). Human immunodeficiency virus (HIV) antibody avidity testing to identify recent infection in newly diagnosed HIV type 1 (HIV-1)-seropositive persons infected with diverse HIV-1 subtypes. Journal of Clinical Microbiology.

[10] De Gruttola V, Lagakos SW (1989). Analysis of doubly-censored survival data, with application to AIDS. Biometrics.

[11] Kim MY, De Gruttola V, Lagakos SW (1993). Analyzing doubly censored data with covariates, with application to AIDS. Biometrics.

[12] Gentleman R, Vandal AC (2002). Nonparametric estimation of the bivariate CDF for arbitrarily censored data. Canadian Journal of Statistics.

[13] Reich NG, Lessler J, Cummings DAT, Brookmeyer R (2009). Estimating incubation period distributions with coarse data. Statistics in Medicine.

[14] Turnbull BW (1976). Empirical distribution function with arbitrarily grouped, censored and truncated data. Journal of the Royal Statistical Society Series B-Methodological.

[15] Maathuis M (2007). MLEcens: Computation of the MLE for bivariate (interval) censored data. http://www.stat.washington.edu/marloes.

[16] Spiegelhalter D, Thomas A, Best NDL (2003). WinBUGS Version 1.4 User Manual.

[17] Gelman A, Hill J (2007). Data Analysis using Regression and Multilevel/Hierarchical Models.

[18] Spiegelhalter DJ, Best NG, Carlin BP, van der Linde A (2002). Bayesian measures of model complexity and fit. Journal of the Royal Statistical Society Series B-Statistical Methodology.

[19] Galli C, Bossi V, Regine V, Rodella A, Manca N, Camoni L, Suligoi B (2008). Accuracy of different thresholds for the anti-HIV avidity index. Microbiologia Medica.

[20] Parekh BS, Hu DJ, Vanichseni S, Satten GA, Candal D, Young NL, Kitayaporn D, Srisuwanvilai LO, Rakhtam S, Janssen R, Choopanya K, Mastro TD (2001). Evaluation of a sensitive/less-sensitive testing algorithm using the 3A11-LS assay for detecting recent HIV seroconversion among individuals with HIV-1 subtype B or E infection in Thailand. AIDS Research and Human Retroviruses.

[21] Parekh BS, Kennedy MS, Dobbs T, Pau CP, Byers R, Green T, Hu DJ, Vanichseni S, Young NL, Choopanya K, Mastro TD, McDougal JS (2002). Quantitative detection of increasing HIV type 1 antibodies after seroconversion: a simple assay for detecting recent HIV infection and estimating incidence. AIDS Research and Human Retroviruses.

[22] McDougal JS, Parekh BS, Peterson ML, Branson BM, Dobbs T, Ackers M, Gurwith M (2006). Comparison of HIV type 1 incidence observed during longitudinal follow-up with incidence estimated by cross-sectional analysis using the BED capture enzyme immunoassay. AIDS Research and Human Retroviruses.

